# Longevity

**Published:** 1894-06

**Authors:** D. E. Nelson

**Affiliations:** Chattanooga, Tenn.


### SELECTIONS.

### Longevity.

BY D. E. NELSON, M. D., CHATTANOOGA, TENN.

Read before the Tennessee State Medical Society, April 10, 1894.

The subject of the paper I bring before you is longevity; a subject that has been a fruitful and profoundly interesting theme of discussion, not only in our profession, but among thinkers and students in every department of knowledge and in every age. Even the comparatively unlettered masses of mankind have not permitted it to pass their observation without their quota of crude, confusing, and altogether irreconcilable opinions and conjectures. There seems to be a peculiar fascination attaching to it, that instinctively challenges attention and stimulates mental activity until our faculties are not satisfied without, at least, a voyage of discovery. Like everything in existence, the deeper it is immersed in mystery, the greater our curiosity to solve it, or, at least, to make the effort, and to satisfy ourselves whether or not it may be a new departure in the course of human events, or a piece of charlatanry utterly unworthy the valuable time and precious thought spent upon it. If it is governed by any specific law, that law is at yet undiscovered. If it is subject to any particular regimen, that also remains hidden.

Is it not surprising that, notwithstanding all that has been written on the subject of longevity, so little is really known of the causes and conditions of long life? Statistics show that a majority of those living to be more than one hundred years old are women. Sir G. C. Lewis, who carefully investigated this subject, positively denied that any *man* ever reached a hundred years, though he was nearly convinced that there were in his day some authentic cases of *female* centenarianism. His argument for his position, was that since the Christian era, no person of royal or noble birth was ever alleged to have reached that magic limit. The lives of the centenarians swell or diminish in length as we advance or recede from prehistoric times. If it be argued that men in high official positions are exposed to greater dangers and more exhausting labors than other men; that the cares of state, the fierce contentions of politics, the brain-work incident to tangled affairs cut short their days, it may be urged on the other hand, that the higher the station the better the medical attendance, the food, air, clothing, and all other conditions on which health and longevity are supposed to depend. It must be admitted that the higher a man's rank the greater is the chance of accuracy in respect to dates; and that if in all the cases which can be easily attested, centenarianism has been found to be a myth, then there is a strong presumption against the obscure centenarians who grow up in places where the system of registration is unknown, and where skepticism is less common than credulity and love for the marvelous. The presumption, however, is liable to be rebutted by facts; and we think they exist in sufficient abundance, not only to overthrow it, but to prove centenarianism beyond a doubt. Flourens was of the opinion that thirty years is the age of man's maturity, and that it marks one-third of his life.

We are justified in saying man has a normal life of ninety to one hundred years, and that by reason of strength he may reach one hundred and ten years. The Biblical expression: "His days shall be one hundred and twenty years," has been thought to mean that this should be the extreme duration of life; others think it meant the average. Judging of the subsequent facts the latter appears to be the correct interpretation. Abraham lived one hundred and sixty-five years. Joshua one hundred and ten. An inscription on an Egyptian monument states the extreme duration of life in that country to be one hundred and ten years. This was the age of Joseph. In the time of David a man of eighty was considered very old. The great prophet king himself reached only seventy-five years. In the ninetieth Psalm, tenth verse, it is said: "The days of our years are three-score years and ten, and if by reason of strength they be four-score years, yet is their strength labor and sorrow, for it is soon cut off and we fly away."

Seventy in the Middle Ages, was considered a great age, which few attained, but it is a fact that in modern times the average is greatly increased. But if normal longevity is thus established, what does science teach as to possible longevity? To what period may exceptional lives extend? May not individuals be endowed with matter of life so largely, and have so slight expenditure, that life may be extended far beyond the limits now fixed by various writers? Is one hundred years the extreme limit of human life, and may we not, under favoring conditions, extend life to variable periods much beyond one hundred years? In the patriarchal age man led a natural life. He lived principally out of doors and took moderate exercise. The tax upon his nervous system was moderate, his food was simple and nourishing, and his digestion consequently good. Accident and old age were then the principal causes of death. At the age of seventy-five, Abraham migrated with all of his possessions. Isaac was born when his father was one hundred and his mother ninety years of age. Abraham was over one hundred and twenty years when he bore the great trial of his faith. Jacob lived one hundred and forty-seven years, notwithstanding his troubles.

To go back to the Romans, Pliny states, from the record of a census taken during the reign of Vespasian, that there were living in the year 76 in Italy, in a certain district, one hundred and twenty-four persons who had attained the age of one hundred years and upward. Haller, long ago declared that over eleven hundred persons had been known to have lived to various ages, between one hundred and one hundred and sixty-nine years.

Thomas Bailey's book, "Records of Longevity," published in 1857, contains the names of about four thousand centenarians, and Dr. Van Owen has collected notices of six thousand, two hundred and two. Of the latter we have the names, country, conditions and date of ninety-nine, who reached the age of one hundred and thirty; of thirty-seven, who lived to be one hundred and forty; of eleven, who reached one hundred and fifty; of seventeen, who exceeded a century and a half. Lord Bacon says that the Countess of Desmond lived one hundred and forty years and cut three sets of teeth. Allen's "American Biographical

Dictionary" gives the names of more than two thousand centenarians; among them are Abraham Bogart, who died in Tennessee in 1833, at the age of one hundred and eighteen, and Francis Age, who died in Pennsylvania in 1767, aged one hundred and thirty-four. Judge Basil Hamilton, of Kalamazoo, Mich., who died a few years ago, aged one hundred and three, was one of a family of twenty-three children, and he himself had seventeen of his own. Dr. Archer Atkinson, in the *Virginia Medical Monthly* for February, 1894, in an article on longevity, says that Lucy Kennon, of Kentucky, was still living in 1876, and aged one hundred and twenty-three years; that Rosetta Washington, of Louisville, Ky., aged one hundred and twenty-one years, was still living in 1888. Stephen Goodale, of York, Maine, died ten years ago, aged one hundred and eighteen. Antoine Save, a native of Normandy, lived to be one hundred and thirty years. Joseph Crele, of Wisconsin, died aged one hundred and forty years. Samuel Stocker, born near Clarksville, Tenn., February 1783, is now living in good health at Toledo, Ohio. George Foster, colored, died in Chattanooga, April 17, 1892, aged one hundred and twelve years. Catherine Patton, colored, is now living in New York city, aged one hundred and eight, and has two daughters living, aged respectively seventy-four and seventy years.

Without considering further the arguments brought forward to establish normal and possible longevity, we may state it as a truth, accepted by scientific men, that man can, to a great extent, control both. The above cases taken together, however incredible some of them may be, seem conclusive. Granting that many of them are open to suspicion, yet after the utmost allowance has been made for errors, misstatements, and wilful exaggerations, enough still remains to establish the truth of ultra-longevity beyond all rational doubt. It is so generally admitted that the duration of life has increased during the present century as to call for no proof. But what are the conditions of long life, so far as can be gathered from the known cases? Happy and calm will be the sleep of those who, on their pillow, can muse on the consolations of the Gospel, and resign themselves implicitly and without wavering into the keeping of a Heavenly Protector and Father. Arbuthnot said: "The instances of longevity are chiefly among the abstemious;" "More men dig their graves with their teeth than with the spade."

Are agricultural districts more favorable than manufacturing; the fresh, open country than the crowded city; mild climates than those whose skies are perpetually scowling? Statistics, well authenticated reports on sickness and mortality, say rural districts have, at most, the advantage of one in two hundred deaths above city districts, and one in five hundred above the town. Against the overcrowding, the bad air, excitement, and the liability to accident in the cities, are set the better water, the greater variety of food, the better knowledge of the laws of health, the more accessible and skillful medical aid, so that the advantages and disadvantages are nearly balanced. The dweller by the sea naturally selects the mountain for his diversion in the summer, and the mountaineer in turn goes to the seacoast, and comes home again in the autumn as to a haven of rest; or if he must labor it is with something like glee that he rejoins his work. After all, we are constrained to say: "There is no place like home." Hot climates have no superiority over cold; China is no more unhealthy than Norway, Iceland or Greenland. Is exercise a vital condition of longevity? Some have said not, in view of the fact that a vicar, cited by the London *Quarterly Review*, Rev. Wm. Davies, reached one hundred and five, though his only exercise for the last thirty-five years was to slip one foot before the other from room to room. Men have lived one hundred years and upward, who only taxed their physical powers to walk one hundred yards a day from house to office and back. Some of the strongest constitutions have been possessed by old soakers. Daniel Bull McCarthy, of Ireland, who drank freely of undiluted rum and brandy during the last seventy years of his life, died in 1752, aged one hundred and eleven. George Kirton, of Yorkshire, a hard drinker, lived to be one hundred and twenty years old. But these are only exceptional cases.

Is a proper diet a *sine qua non* of longevity? All writers on health denounce newly made bread, and especially hot bread, and not a few denounce tea and coffee; but can not each of us call to mind the names of very old people who have been for the greater portions of their lives free users of the same? Shall we declare in favor of frequent bathing or personal cleanliness? Animals get rid of the outer layer of skin in some way. In some species, certain reptiles for example, the outer skin is shed entire at certain seasons. Birds cast their feathers, and fish their scales; and at each casting off of his kind, the vital processes are afterwards carried on with more energy, because the drains and sluice-ways of the surface are not blocked up at their mouths by broken down epidermis; so in man, the skin is removed; only the process in a state of health is gradual and less startling in its visible signs. What shall we say of "Lady" Lewson, a London widow, who never washed her rooms, nor bathed, and declared that people who bathed were constantly taking cold; but she habitually smeared her face and neck with hog's lard. It is said of the Icelanders, that though they are very uncleanly and suffer much from skin diseases, especially leprosy, their longevity is greater than that of the nations of Europe.

The one great question of sanitary science is to secure to each individual, death from old age. On every hand we witness the prodigal waste of human life. Of the children born, what a vast percentage never see the anniversary of their birth! What a large percentage die under five years of age! And how few, comparatively, reach the age of ten! At twenty the generation has dwindled to an insignificant minority, and at thirty-three to forty-five disappears altogether. The science of life reveals to us the stupendous fact that man is born to health and longevity, and that disease is abnormal, and death, except from old age, is accidental, and that both are largely controllable by human agencies. We find that physiology teaches that in every tissue and in the corporate whole, there is inherent death.

It is evident, therefore, that the question of longevity is to be determined by estimating, correctly, vital endowment and vital expenditure. In man, the tendency to individuation is strongest and the degree of evolution is the highest. Man being omnivorous, can secure his food from many sources. He can sustain vigorous health on the products of the soil, or the river, or the forest. Nor need he expend his strength unavailingly in search of food, for by his ingenuity he can devise means of securing it with but slight effort. By suitable protection, man may and does husband his native resources and reduce to a minimum the expenditure of his vital forces. Consider also, by what skillful appliances he continues to repair the wastes and inroads of decay or accident upon his bodily organs and functions. It is a fact that the average length of human life has gradually increased in many civilized communities.

In 1665, in London, one twenty-third of the people of that city died, but now the rate of mortality has been reduced to one forty-fifth annually. Is this not proof of man's advance in civilization, which enables him to live with less expenditure of vital force, which leads him to see his own highest welfare in the common effort to promote the advance of sanitary science?

Mr. Mathews says that health is not a condition of long life, and that long life is no more dependent upon health than upon great muscularity. He cites as proof that the Tom Hyers and Heenans, great prize fighters, and heavy weight lifters, who could fell an ox with their fists, were always ailing, and rarely lived to be more than fifty or sixty years old; and that Dr. Winship, of Boston, who could lift three thousand pounds, died aged forty-two. The celebrated Galen had a weak and delicate constitution, yet by strict temperance and evenness of temper, lived one hundred and forty years. It is said that his rule in eating was to rise from the table before his appetite was satisfied. Dr. Southy, in the London *Lancet*, ten years ago, said that the three oldest people he ever knew were women, who reached respectively, eighty-nine, ninety-eight, and one hundred years, and were nearly all their lives of infirm health. And thus I might go on and cite numerous other such cases, but this is enough to bring within the scope of this paper.

In spite of all these facts, it is hard to believe that virtuous habits, abstemiousness, exercise and cleanliness do not conduce to longevity. But the one thing that outweighs all other favorable circumstances is what is called "a certain bodily and mental predisposition to longevity." Sir Thomas Brown wrote: "There are persons who are prefigured unto a long duration." Some persons are born with a genius for long life, but it is a gift which can be cultivated, and like any other, it may be destroyed. Those who have this inherited gift will commonly reach old age, though they trample on the laws of health; while those who have it not will die comparatively young in spite of the strictest precaution against disease. How is it that in a race of beings having such a power over its destiny, and striving by every means to obtain long life, but three in every thousand dying reach their pre-ordained longevity? Within one's own observation he sees a vast array of causes abridging life. In his own make-up he sees sickness on a perpetual warfare with his vital forces.

According to the last United States census, nervous and digestive troubles cause nearly one-third of the deaths. Every point of man's system is subject to attack from disease. The earth and air teem with the germs of disease; we eat them and we breathe them. It is believed by both civilized and savage nations that disease is the expression of the Almighty's disapprobation of man's immoral conduct; and granting this to be true, may we not add that it is the penalty inflicted for the disregard of that Biblical precept: "Cleanliness is akin to godliness?" The most destructive diseases are of man's own creation. The principal condition which shortens life is, first, and above all others, the expenditure of vital force; and it is scarcely necessary for me to mention the various ways by which the vital force may be expended. At maturity the passions ripen into full activity, and have their full play, and if not held under control they rapidly exhaust the vital strength. Man may yield to all his passions and appetites, and perish at maturity, or be decrepit at thirty. He may so order his life as to fully and healthfully develop his passions and appetites so that they shall culminate in a prolonged and vigorous old age, and thus leave death as a subject of accident. It is an axiom of enlightened statesmanship, that the great want of any country is, and ever will be, a healthy, robust, and long-lived yeomanry. Public health means public wealth, honesty and morality. Our government can in no way better promote the interests of the people than by diffusing knowledge on these points.

One of the most sensible sayings on the art of longevity that has fallen under my notice, so far as longevity can be considered attainable, was that given by an Italian in his one hundred and sixteenth year. Being asked the secret of his living so long, he replied;

> "When hungry, of the best I eat,
> And dry I keep my feet;
> I screen my head from sun and rain,
> And let few cares perplex my brain."

In these lines. especially the last, we have the quintessence of all the advice that has been or can be given on the subject. The deadliest foe to longevity is excitement. "To live long," said Cicero, "is to live slowly."

The careful stock raiser shows his faith in heredity by strict examination of the pedigree; and why so well demonstrated, a great physiologic truth escapes notice when a human being is concerned instead of a horse, is in the nature of a conundrum to all thoughtful minds. He who lives extensively, who avoids all stimulants, takes light and agreeable exercise, indulges no exhausting passions, feeds his mind and heart with no exciting material, has no debilitating pleasures, "keeps his accounts with God and man daily squared up," is sure, if he has a good organism, to spin out his life, barring accidents, to the longest possible limit.

The poet, William Cullen Bryant, when asked the secret of his health and vigor at upwards of eighty, answered; "It is all summed up in one word, *moderation.*" The ancients laid much stress on gastronomy, as many idioms in all languages attest. "Heaven sends the food, but the devil sends the cook," is an old saw that is more forcible than elegant. How many a young man squanders on a holiday or an evening's entertainment an amount of nervous energy which he will bitterly feel the want of when he is fifty. It is true that overwork of the eye at fourteen may necessitate spectacles at forty, instead of at sixty. Even warm affections are prejudicial; they subject the owner to constant anxiety, and are as unnerving as the excitement produced by politics or gambling. Nothing is more exhausting than anxiety for a sick wife or child, or nursing a friend through a long sickness, unless you can truthfully say that you take no interest in the result. When a "fine old man" was mentioned in Swift's presence, he exclaimed angrily: "There's no such thing; if his head or heart had been all right they would have worn him out long ago." Wadsworth at eighty, complained:

> "Oh, but the good die first
> And we whose hearts are dry as dust,
> Burn to the socket."

Our fine enthusiasms, if they rise into intensity, are costly, and lessen the number of moments we have to live. Some years ago a gentleman in England set about ascertaining the causes of the premature deaths of his acquaintances who had been cut off within twelve years. Of forty individuals, he found that twenty had died from excessive mental labor or excitement, and twelve of these were not intellectual laborers, but men of the world. Sydenham tells us that one of the severest fits of gout he ever suffered from, arose from great mental labor, undergone in composing his treatise on that disease.

Providence has appointed the succession of labor and rest by the alternation of day and night, yet how many violate this beneficent law by turning night into day and day into night. They sleep while the sun is shedding his life-giving beams, and work amid the deadly influences of the night. Many do not toil at their callings in the night-time, yet imagine that they do a full day's work, and afterwards, with impunity, spend half of the night in charitable labor, or in the pursuit of pleasure or knowledge. But nature can not be so cheated. Occasionally she lets an offender escape for forty or fifty years, but she is evermore "shadowing" him, and hauling him up at last inflicts her penalty just when and where he least expects it. It should be remembered that one can walk farther than he can run.

While all excess is injurious, it must not be inferred that hard brain-work apart from other causes tends to shorten life. Mental labor, apart from grief and fears, from forced or voluntary stinting of the body's needed supply of exercise, food or sleep, and the mind's supply of social intercourse, rather prolongs life than cuts it short. Even overwork of the brain is probably far less injurious than underwork. Nine-tenths of the students and professional men who are supposed to break down from intense toil, wear themselves out, not by repletion of study, but by a vicious misapplication of their moral, physical or intellectual forces.

Lord Brougham lived eighty-nine years. Lord Lyndhurst wore out at ninety-one. Epimenides, the seventh of the wise men, is said to have lived to one hundred and eighty-four. Hippocrates reached ninety-nine, Pythagoras reached eighty, and was murdered. Dr. Franklin was eighty-four. Montgomery, the poet, lived to be eighty-two. Sydney Smith lived to seventy-six, and Sir Isaac Newton to eighty-five. The Right Hon. William E. Gladstone, who retired from the Premiership of England only a short time ago, was last December, eighty-four years old. Dr. Beard, of New York, in an able paper on the "Longevity of Brainworkers," has proven beyond even the shadow of a doubt that the world's hardest workers, so far from being short-lived, show a very high average of life; a far higher average than the world's drones, and those who had added nothing to its accumulated capital of happiness, knowledge, goodness and truth. It is an established fact that not a few of the long-livers might materially lengthen their days by taking more exercise and sleep, and by economizing more carefully the expenditure of intellectual and moral energy.

It is known by every scholar that mental application is one of the most effective means of relieving bodily pain, and that it is especially fitted to soothe the ruffled spirit, and to mitigate the asperity of corroding anxiety and care. Bacon, in his "History of Life and Death," is emphatic in declaring the religious and literary to be among the forms of life most conducive to longevity. "There are," says he, "in religious life, leisure, admiration and contemplation of heavenly things, joys not sensual, noble hopes, wholesome fears, sweet sorrows, continual renovations by observances, penances and expiations, all of which are very powerful to the prolongation of life." There is a popular notion, which has long been deeply rooted, that precocity of intellect is unfavorable to longevity. It has been shown conclusively, that as a rule, a brain of exceptional force is united to a constitution of exceptionally good fiber, and that precocity, so far from being premonitory of early decay and death, is almost a mark of vast undeveloped abilities, and of a prolonged existence crowded with triumphant usefulness.

Three of the most precocious geniuses of their day were Bishop Thirlwall, Lord Macaulay and DeQuincy, yet they lived to the ages respectively of seventy-eight, fifty-nine and seventy-four. Of all the qualities of mind that conduce to longevity, none are more vitally essential than contentment, cheerfulness and hope. Worry kills more men than the most exacting work, whether physical or mental. Legitimate work develops force, while worry checks its development and wastes what already exists. The physician should be engaged to protect the household and individuals from the assaults of disease, to detect and meet the first and very slightest indications of disorder; and thus we shall certainly see life prolonged.

There is no doubt that long life, if it be virtuously and happily spent, is a blessing most earnestly to be coveted. The mere lapse of years, however, is not life; "knowledge, truth, love, beauty, goodness and faith give vitality to the mechanism of existence." The value of time is purely relative; and, if we count it by heart-beats, not by tickings of the clock, or the shadow of the dial; if, "he lives longest," as Bailey says, "who knows the most, thinks the wisest, acts the best;" then many who were rich in years have really died young, while others whose lives measured by the calendar, were cut short early, have been rich in life.

Shakespeare, who died at fifty-two, lived ten times as long as poor old Parr, who could boast of his one hundred and fifty-two years. Mere old age, following an oyster-like existence, during which one has droned away his life in his shell, never buffeting the waves for himself or others is a questionable blessing; but the serene old age which is secured by temperance, sobriety, and the conquest of vicious appetites and passions, the long, mellow autumn of life, in which are harvested the fruits of years of useful toil, is to be coveted and striven for by all.

> " Men deal with life, as children with their play,
> Who first misuse, then cast their toys away."

*Jour. of the Am. Med. Assn.*

---

THE INTERNATIONAL CONGRESS OF MEDICINE, held at Rome, received a petition signed by more than seven hundred doctors in India. This petition demands that Latin shall henceforth be the universal scientific tongue, and that a central medical bureau be established, charged with the translation into Latin of all modern works worthy of preservation; also the creation of a medical journal published in Latin, this publication to be called *Salernum,* in memory of the first school of medicine in Europe, founded at Salerno. A society known as the " Ancient Order of Æsculapians" should also be organized ; one of the conditions of admission being an original thesis written in either Latin or Greek. The names of Hippocrates and Galen will survive even after future centuries, when the Kochs and Pasteurs *et id genus omne* are forgotten or buried in the chaos of medical oblivion. Modern surgery will survive, but modern medicine, the essence of quackery and pretension, tinctured by the commercial spirit of a rotten age, will perish. So, after all, there will be little that is modern worthy of being presented in the classical tongue, and the task of translation would be a light one. The advent of the new journal, *Salernum,* will be welcomed by those who are worthy of fellowship with the " Ancient Order of Æsculapians."

*The Cincinnati Lancet-Clinic.*

---

A HOSPITAL OF LEPERS, with a laboratory for the study of leprosy, has been established at Rio de Janeiro. Dr. Wolf Havelburg, a German physician, who for the last twelve years has practiced in Brazil, has been appointed director.—*Medical Review.*

